# Simply Adding Oxygen during Hypothermic Machine Perfusion to Combat the Negative Effects of Ischemia-Reperfusion Injury: Fundamentals and Current Evidence for Kidneys

**DOI:** 10.3390/biomedicines9080993

**Published:** 2021-08-11

**Authors:** Tom Darius, Jay Nath, Michel Mourad

**Affiliations:** 1 Surgery and Abdominal Transplant Unit, University Clinics Saint Luc, Université Catholique de Louvain, 1200 Brussels, Belgium; michel.mourad@uclouvain.be; 2 Pole de Chirurgie Expérimentale et Transplantation, Université Catholique de Louvain, 1200 Brussels, Belgium; 3 Department of Renal Transplantation, Southmead Hospital Bristol, Bristol BS10 5NB, UK; jay.nath@nhs.net

**Keywords:** hypothermic machine perfusion, kidney transplantation, oxygenation, mitochondria, organ preservation, kidney assessment

## Abstract

The use of high-risk renal grafts for transplantation requires optimization of pretransplant preservation and assessment strategies to improve clinical outcomes as well as to decrease organ discard rate. With oxygenation proposed as a resuscitative measure during hypothermic machine preservation, this review provides a critical overview of the fundamentals of active oxygenation during hypothermic machine perfusion, as well as the current preclinical and clinical evidence and suggests different strategies for clinical implementation.

## 1. Introduction

The donor organ shortage remains one of the major challenges in kidney transplantation with the consequence that many patients still die whilst awaiting a transplant [1]. As a result of this shortfall, higher risk organs such as those originating from expanded criteria donors (ECD) of brain death donors (DBD) or donated after circulatory death (DCD) are nowadays more frequently utilized. These kidneys are more susceptible to ischemia-reperfusion injury (IRI), which results in higher risk of delayed graft function (DGF), primary nonfunction (PNF), and graft failure [2,3,4,5]. The damage to the podocytes in the tubulus, also known as acute tubular necrosis, mainly caused by IRI, is considered the main cause of DGF after transplantation [6]. Unfortunately, a significant number of kidneys are discarded because of the lack of objective criteria to assess organ quality in the pretransplant period and perceived limitations of organ resuscitation and repair during preservation [7,8,9,10,11,12]. Over the past twenty years, machine perfusion strategies have gained greater clinical traction to improve organ preservation, viability assessment, and organ utilization, and to decrease the harmful effects of IRI. A variety of different perfusion techniques have been described but the most widely used are normothermic and hypothermic machine perfusion (HMP).

Normothermic machine perfusion (NMP) provides a near-physiological environment which offers an opportunity for ex-vivo kidney evaluation (quality assessment) and has the potential to decrease the harmful effects of ischemia-reperfusion injury after cold preservation [13,14] and even few ischemia-free transplantations are reported [15,16]. End-ischemic normothermic perfused kidneys was demonstrated to result in a significant reduction in DGF rate (36.2% versus 5.6%, *p* = 0.014) as compared to static cold storage (SCS) alone [17]. Recruitment of a first multicenter randomized controlled end-ischemic NMP UK-trial has recently terminated and results are expected soon [18]. In contrast with normothermic perfusion, which requires blood-based oxygenation or other oxygen (O_2_)-carrier, as well as attention to nutrient supply, acid-base balance and disposition of products of metabolism, HMP is less complex and cheaper. In addition, there is no risk of organ loss in the event of pump failure. For these reasons, HMP is the most established perfusion technique for deceased donor kidneys. A recent meta-analysis highlighted improved outcomes for kidneys compared with SCS, with reduced rates of DGF and PNF and superior one-year graft survival (OR:1.61 95% CI: 1.02 to 2.53, *p* = 0.04) [6]. This meta-analysis is in line with the meta-analysis of Tingle SJ et al. showing superiority of HMP to SCS for both DBD and DCD kidneys [19]. As kidneys from DCD donors have a higher overall DGF rate, fewer perfusions are needed to prevent one episode of DGF (7.26 versus 13.60 in DBD kidneys) [19].

Oxygen consumption in hypothermic conditions (4 °C) is reported to occur at approximately 5–10% of that at normal body temperature [20,21,22]. Even under the highly non-physiological conditions of HMP, kidney oxygen consumption has been demonstrated, with a reported 90% decrease in perfusion fluid oxygen levels after 2 h [23]. Given this oxygen consumption by the perfusion kidney, additional oxygenation could further promote oxidative processes within the organ, restoring the adenosine triphosphate (ATP) debt and decreasing the ischemic accumulation of mitochondrial succinate responsible for the harmful effects of IRI. However, the addition of oxygen during HMP could also potentially increase the production of radical oxygen species (ROS) and thereby cause increased injury [21]. 

With oxygenation as an additional resuscitative measure during HMP preservation, this review provides a critical overview of the fundamentals of active oxygenated HMP, preclinical and clinical evidence and proposes different strategies for clinical implementation.

## 2. Hypothermic Machine Perfusion

The concept of HMP is not new and early research in this field dates back to the 1960s [24,25]. Belzer designed the first clinical machine perfusion device used during the first successful human transplantation of a 17-h hypothermically machine-perfused kidney in 1968 [26]. HMP preserves the graft by a continuous or pulsatile administration of a recirculating cold preservation solution (1–10 °C) which results in a continual flush of the microcirculation and prevents the accumulation of toxic metabolites. The most commonly perfusion fluid is the University of Wisconsin solution modified for machine perfusion. This is an acellular hypertonic fluid without a designated oxygen carrier. Numerous portable devices are commercially available to deliver HMP including the LifePort Kidney Transporter (Organ Recovery Systems, Itasca, IL, USA), the Kidney Assist Transporter (Organ Assist BV, Groningen, The Netherlands) and the WAVES machine (Institut Georges Lopez, Lissieu, France). The RM3 device (Waters Medical Systems, Rochester, MN, USA) is a not portable but used widely in the USA. The VitaSmart (Bridge to Life, Northbrook, IL, USA) is a multi-organ not portable device to implement end-ischemic hypothermic oxygenated perfusion. One of the primary differences between devices is that some are pressure-driven (e.g., LifePort and Kidney Assist), whilst others are flow-driven (e.g., RM3 system). The superiority of one machine over the other remains a matter of debate. Some evidence shows that HMP should be controlled by pressure and not flow, using low pressures to avoid pressure-related injury [27,28]. A pulsatile renal artery pressure of 25–30 mmHg is the best for the kidney [29,30,31]. Whilst some HMP devices incorporate oxygenation as standard, all devices can be easily modified to incorporate an oxygenator. 

The exact working mechanism of HMP is multifactorial and based on mechanical vasodilation, enhanced endothelial nitric oxide synthase phosphorization and molecular vasoprotection (decreased vementin, fibrosis, endothelin, innate immunity, Toll like receptor 4, High Mobility Group Box 1 (HMGB1), cytokines, and increased protective endothelial genes, hypoxia-inducible factor-α and nitric oxide signaling) [32,33,34,35,36,37,38,39,40]. 

The main focus of current research to decrease subsequent kidney IRI during HMP is situated on 4 levels: (a) cellular therapy (e.g., mesenchymal stem cells), (b) pharmacological therapy and biologicals (e.g., curcumin [41], antioxidants (trimetazidine [42], cyclodextrin [43]), artificial oxygen carriers [44]), (c) gene therapy and (d) addition of gas. This review focus on the role of oxygen to protect the kidney against IRI during HMP.

## 3. How to Deliver Oxygen to the Kidney during HMP?

The optimal oxygen administration technique during HMP has not yet been established but currently two techniques exist for clinical application: (1) membrane oxygenation and (2) bubble and surface oxygenation. 

### 3.1. Membrane Oxygenation 

**Figure 1:**
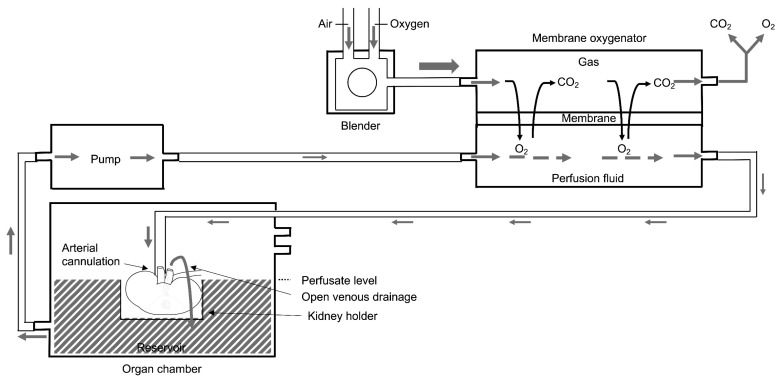
Basic principles of membrane oxygenation during kidney perfusion. Oxygen diffuses from the gas into the perfusion fluid, and carbon dioxide diffuses from the perfusion fluid into the gas for disposal. The oxygenated perfusion fluid is pumped into the kidney by arterial cannulation. Via open venous drainage, the perfusion fluid is collected into the reservoir of the organ chamber and again pumped towards the membrane oxygenator to reoxygenate the perfusion fluid.

Hollow fiber membrane oxygenators have replaced bubble and classical membrane oxygenators as extracorporeal oxygenators in most clinical applications (e.g., cardiac surgery) from the beginning of the 1980s. This was due to superior gas exchange, compared with classical membrane oxygenators and reduced haemolysis compared to bubble oxygenation [45,46]. Because of their use in other clinical settings, hollow fiber membrane oxygenators were incorporated in the commercially available Kidney Assist Transporter (Organ Assist BV, Groningen, The Netherlands) and the VitaSmart (Bridge to Life, Northbrook, IL, USA) but also incorporated in “home-made” non-transportable models, as recently illustrated in an Italian trial [47]. The principles of membrane oxygenation and general perfusion set up are illustrated in Figure 1. A membrane oxygenator is added in series to the perfusion set up between the pump and the organ chamber and consists of a thin gas-permeable membrane separating the perfusion fluid and gas flow. Oxygen diffuses from the gas into the perfusion fluid, and carbon dioxide diffuses from the perfusion fluid into the gas for disposal. The oxygenated perfusion fluid is pumped into the kidney by arterial cannulation. Via open venous drainage, the perfusion fluid is collected into the reservoir of the organ chamber and again pumped towards the membrane oxygenator to reoxygenate the perfusion fluid. 

The oxygenated Airdrive HMP system (Doorzand, Amsterdam, The Netherlands) is a new portable, disposable device and perfuses the organ by means of an oxygen pressure-driven membrane pump [48]. A hollow fiber membrane oxygenator in the machine perfusion circuit allows for active oxygenation of the preservation solution to achieve partial oxygen pressure (pO_2_) of at least 80–100 kPa (600–750 mmHg) [49,50,51].

### 3.2. Bubble and Surface Oxygenation

**Figure 2:**
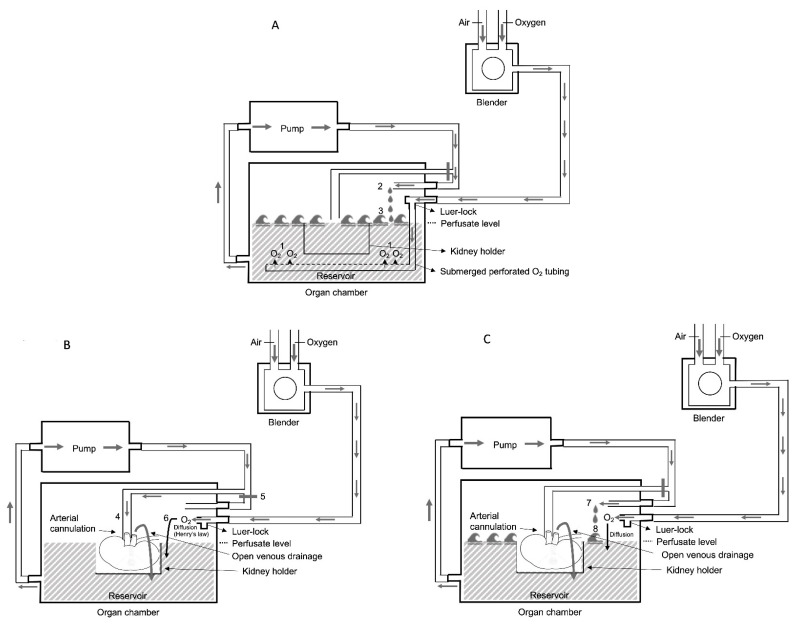
Basic principles of bubble and surface oxygenation during kidney perfusion. (**A**) Bubble oxygenation before connecting the kidney to the device via the submerged perforated O2 tubing segment (1), and the perfusion fluid enters the reservoir by a separate wash line (2) and by creating waves the efficiency of this O2 uploading process increases (3). (**B**) Surface oxygenation during kidney perfusion via arterial cannulation (4) (and closure of the wash line (5) and removing of the submerged tubing segment of the Luer-lock (6)). (**C**) The efficiency of surface oxygenation is enhanced during regularly scheduled wash cycles (7) resulting in breaking the perfusate’s surface layer (8) and therefore increasing oxygen diffusion in the perfusate.

Whilst membrane oxygenation is undoubtably effective at oxygen transference, it is likely to be well in excess of the kidney requirement for sustaining aerobic metabolism of a single kidney at ±4 °C during HMP. For example, the average O_2_ transfer of 14.8 mL O_2_/min of the Dideco kids D100 neonatal oxygenator [52] far exceeds (by over twenty-fold) the O_2_ consumption of a 100–200 g kidney at 4 °C which is estimated at 0.68 mL O_2_/min. [53] Therefore, there has been renewed interest in bubble and surface oxygenation to raise the dissolved perfusate O_2_ concentration. This technique is based on four principles: First, bubble oxygenation (before connection of the kidney to the device) is directly proportional to the oxygen volume and inversely proportional to the bubble size. This results in a highly effective O_2_ transfer (achieving also perfusate pO_2_ of at least 80–100 kPa (600–750 mmHg) after 20 min of bubble oxygenation [54]. Although continuing bubble oxygenation of the perfusate during kidney perfusion would have been highly effective to maintain supraphysiological pO_2_ levels, it should be halted since it could cause massive foam formation due to protein degradation during kidney perfusion. Secondly, the solubility of oxygen increases as temperature decreases [44,55]. Thirdly, according to Henry’s law, oxygen will slowly diffuse across the surface of the perfusate from the gaseous compartment on the top of it. The amount of O_2_ diffusing into the perfusate will be proportional to the percent (=partial pressure) of O_2_ above its surface [56]. Fourthly, the efficiency of surface oxygenation is enhanced during the regularly scheduled wash cycles (every 10 min) during machine perfusion resulting in breaking the perfusate’s surface layer and therefore increasing oxygen diffusion in the perfusate (comparable how oxygen enters the water in open sea by wind and waves). This alternative oxygenation technique, applied to the perfusion kits of the LifePort Kidney Transporter (Organ Recovery Systems, Diegem, Belgium) was proven to be as effective as membrane oxygenation to achieve supraphysiological perfusate oxygen concentrations (needed to trigger ATP resynthesis as described by Lazeyras et al. [57]) in a pig kidney autotransplant model and now available for clinical implementation [58] and is illustrated in Figure 2. Oxygen uploading of the perfusate is obtained by bubble oxygenation in the reservoir during the wash phase during set-up before connecting the kidney to the device. This is realized by insufflating the carbogen directly to the perfusion solution in the reservoir via the submerged perforated O_2_ tubing segment. During wash phase the perfusion fluid enters the reservoir by a separate wash line and by creating waves the efficiency of this O_2_ uploading process increases (Figure 2A). Once the kidney is connected to the device and perfused via arterial cannulation and closure of the wash line, bubble oxygenation is switched to surface oxygenation (after removing of the submerged tubing segment of the Luer-lock). The perfusion fluid is collected by open venous drainage into the reservoir where oxygen will slowly diffuse across the surface into the perfusate and afterwards pumped again in the direction of the arterial cannule (Figure 2B). The efficiency of surface oxygenation is enhanced during regularly scheduled wash cycles (every 10 min) during machine perfusion resulting in breaking the perfusate’s surface layer and therefore increasing oxygen diffusion in the perfusate (Figure 2C).

## 4. How Active Oxygenation during HMP Reduces Ischemia-Reperfusion Injury

**Figure 3:**
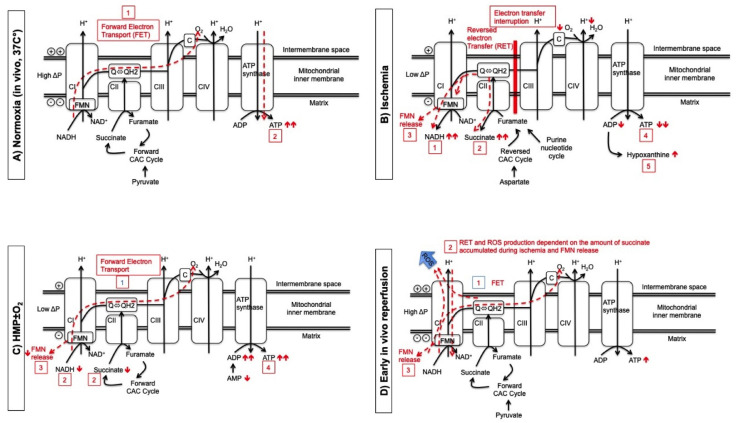
The effect of active oxygenation during HMP on mitochondria through the process of kidney preservation and transplantation. (**A**) During normoxia, forward electron transport (dotted line) (1) drives the proton pumps (CI, CIII, CIV) against a charge gradient at the oxidative phosphorylation chain and proton back flow drives ATP synthase to generate ATP (dotted line) (2). (**B**) In the absence of oxygen, electron transfer is interrupted and first NADH (1) and second citric acid metabolite succinate (2) increase because both act now as electron carrier to allow CI continuously pump protons during ischemia. Also FMN release (3) at CI results from over-reduction of flavin via RET. Consequently, ATP and ADP decrease (4) and purine metabolites increase (5). (**C**) The degree of FET during HMP depends on the perfusate oxygen concentration (1) and results in a decrease of succinate and NADH (2) and less FMN release (3) and an increase of ADP/ATP regeneration (4). (**D**) During early, in vivo reperfusion, FET (1) generates ATP. Rapid consumption of accumulated succinate generates too much QH2, which impairs further FET. In combination with acidic pH from ischemia this results in a RET at CI (2). Reduced or semi-reduced FMN (3) located at the nucleotide-binding site is the direct source of ROS in CI during RET but reduced FMN can also non-enzymatically react with molecular oxygen producing ROS.

Mitochondrial succinate accumulation originating from the citric acid cycle (CAC) during ischemia is one main contributor for superoxide generation by reverse electron transport (RET) during subsequent in vivo reperfusion under normothermic conditions [59,60,61]. Dutkowski et al. described this mechanism assessed during different human liver perfusion strategies [62]. The existence of similar pathological processes during kidney perfusion was confirmed by Darius et al. [63]. The effects of active oxygenation during HMP on mitochondria, through the entire process of kidney preservation and transplantation, are illustrated in Figure 3. The main source of in vivo cellular ATP, during physiologic oxygenated conditions at 37 °C (before procurement of the kidney), is mitochondrial oxidative phosphorylation, which is dependent on oxygen as the final electron acceptor (Figure 3A) [21,55,61,64,65,66]. Complex I (CI), composed of 45 subunits, contains one molecule of tightly, but non-covalently bound flavin mononucleotide (FMN) and eight different iron-sulfur clusters [65]. Flavin serves as the primary electron acceptor from reduced nicotinamide adenine dinucleotide (NADH), while a series of iron-sulfur clusters provide a pathway for the forward electron transfer to the bulk ubiquinone (Q) [65]. During ischemia and hypoxia, forward electron transport (FET) is interrupted with a number of pathological sequelae due to RET (accumulation of NADH, succinate and purine metabolites, FMN release (FMNH2) and ATP/adenosine diphosphate(ADP) decrease)(Figure 2B) [61,62,65,66,67]. As mentioned above, cold machine preservation solutions were originally developed for HMP without active oxygenation and do not contain an oxygen carrier. Therefore, tissue oxygenation during HMP is provided by diffusion. Under oxygen supplemented conditions, the diffusion of oxygen into the HMP perfusion fluid promotes physiological mitochondrial processes with evidence of FET activity. This is evidenced by lower NADH and succinate levels and subsequently less FMN release with greater ATP re-synthesis, compared with standard HMP or end-preservation O_2_ supply during HMP (MODE 3 of mitochondrial operation according to Murphy, Figure 3C) [58,62,63,66]. During early in vivo reperfusion, FET generates ATP in the presence of oxygen. However, rapid consumption of accumulated succinate generates excessive Ubiquinol (QH2), which impairs further FET. Such high succinate levels in combination with an acidotic milieu during ischemia results in RET at complex I with subsequent ROS production (Figure 3D) [61,68]. Recent literature suggests that it is not the accumulated ischemic succinate but more likely, the reduced or semi-reduced flavin located at the nucleotide-binding site, that appears as the direct source of ROS production at complex I [65].

## 5. Preclinical Evidence for Active Oxygenated HMP (HMPO_2_)

Lazeyras et al. observed different energetic states of cold preserved organs taken in consideration HMP and oxygenation [57]. The presence of enough O_2_ (100 kPa) is necessary for ATP resynthesis. In the absence of active oxygenation, Kaminski et al. demonstrated that kidneys following a period of warm ischemia demonstrated a greater consumption of both ATP and oxygen during HMP compared with SCS [69]. This highlights the metabolic upregulation that occurs during HMP [70]. In a study of discarded human kidneys with oxygenation (hyperbaric or normobaric, Ravaioli et al. demonstrated a net increase of ATP following oxygenated HMP [71]. Porcine studies assessing effects of oxygenation frequently utilize either an ex vivo normothermic reperfusion model or an autotransplant model to determine the functional impact of oxygenation. A literature overview of these studies is summarized in Table 1 [34,49,58,63,72,73,74,75,76]. Interestingly, the ex vivo normothermic reperfusion studies demonstrate conflicting results. This might be related to the different preservation and ex vivo reperfusion times and limitations related to the ex vivo model itself. For the autotransplant models, the functional benefits of oxygen are consistent following the ischemic insult, with comparable functional benefits reported following 30 or 60 min of warm ischemia (WI) prior to HMP. In the absence of preceding warm ischemia no benefit of active oxygenation was observed [73]. 

Studies suggest that high perfusate oxygen concentrations results in superior mitochondrial protection and improved initial graft function during continuous HMP strategies as compared to low perfusate oxygen concentration or in the absence of active oxygenation [49,74,76]. The duration and start point of oxygenation during HMP is discussed in more detail in a subsequent section of the review, as is the different methods for providing supplemental oxygen.

## 6. Clinical Evidence for HMPO_2_

The experience of active oxygenation during HMP is currently limited to five clinical trials, with a variety of oxygen preservation strategies, detailed in Table 2 [47,48,50,51,77]. 

The principal evidence to support active oxygenation during HMP is derived from a multicenter RCT from DCD, category III, over 50 years. In this double blinded study of 106 kidney pairs, patients were randomized to receive a kidney following eighter oxygenated or non-oxygenated HMP which was performed during the entire preservation period. Although the study failed to demonstrate an improvement in the primary outcome of 12-month estimated glomerular filtration rate (eGFR), there were several significant findings in the study including reduction in biopsy proven acute rejection (BPAR) and severe postoperative complications (Clavien-Dindo grade III or more) compared with standard HMP without active oxygenation [50]. More pertinently, the graft failure rate was lower in the oxygenated group (3% versus 10%; hazard ratio 0.27, 95% CI 0.07–0.95; *p* = 0.028) [50]. 

These positive findings were not replicated in two matched-case studies and one RCT where oxygenated HMP was performed as an end-ischemic preservation strategy for ECD DBD kidneys. Under such conditions, there was no demonstrable improvement in DGF, functional DGF and PNF rate nor early graft function (at 6 months or 1 year after transplantation) and BPAR rate as compared to SCS alone [47,51,77].

## 7. Mechanical Perfusion Parameters and Biomarkers as Predictors of Outcome

### 7.1. Mechanical Perfusion Parameters

In the United states, more than 15% of machine-perfused kidneys are discarded annually, partially based on elevated renal resistance [78,79,80,81]. However, the only prospective study to determine the true value of the renal resistance during HMP is the Eurotransplant machine perfusion trial [82]. In this trial, the preservation method was not revealed at the time of the organ offer and in case of HMP, the kidney was accepted only based on donor data because renal resistance was unknown to the clinician. Renal resistance at the end of HMP was an independent risk factor for the development of DGF (adjusted odds ratio (AOR) 38.1 (1.56–93.4) (*p* = 0.03)). However, the predictive power of renal resistance was low (area under the curve of the receiver operating characteristic curve 0.58). This means that renal resistance has a limited value to predict DGF and cannot be used on its own to predict graft viability and accept or discard kidneys on HMP. On the other hand, the Montreal group showed that a renal resistance <0.2 resulted in a 5-year graft survival of 93% which might render renal resistance useful as re-assurance tool for accepting marginal kidneys due to its reasonably faire negative predictive value [83]. Recent date from the Milan group indicated for the renal resistance trend is more predictive of post-transplantation outcome than pre-transplantation biopsy evaluation, suggesting that renal resistance to be a less invasive assessment tool better reflecting cortical status [84,85].

### 7.2. Value of Perfusate Biomarkers

The viability assessment in the circulating perfusate have been extensively reviewed in recent publications [32,86,87,88]. The most commonly reported perfusate analysis are also analyzed in the above mentioned Eurotransplant machine perfusion trial and demonstrated that glutathione-S-transferase, N-acetyl-β-D-glucosaminidase and heart-fatty acid binding protein were independent predictors for DGF but not for PNF and graft survival [89]. Lactate dehydrogenase, aspartate aminotransferase, and alanine-aminopeptidase had no independent prognostic value. A systematic review by Guzzi et al. identified glutathione-S-transferase as the most promising biomarker for predicting DGF, but its predictive ability was at best moderate [90]. Rapid metabolomic analysis in the perfusate by nuclear magnetic resonance demonstrated in a preclinical model, that the levels of several metabolites during HMP could be an interesting tool to access graft quality and functional recovery [91]. Also microRNAs are becoming increasingly important as biomarkers in perfusion fluid to predict organ viability [92]. The main disadvantage of most of these markers is their limited predictive value of kidney outcomes and clinical implementation in daily practice. In particular, when applied to assess organ transplantability. In contrast, reduced flavin (FMNH_2_) can non-enzymatically react with molecular oxygen and generate ROS and oxidized flavin, which may rebind at mitochondrial complex I and recover the enzymatic activity during later reperfusion [65]. FMN determination during HMP by simple fluorescence is far more easy, faster and cheaper than any currently available succinate measurement and could therefore potentially be used as real time surrogate marker to assess the metabolic status and predict ischemia-reperfusion injury in solid organs. Indeed, Muller et al. described the use of FMN to predict liver function during hypothermic oxygenated liver perfusion before implantation [93]. FMN detection in the perfusate demonstrating significantly lower values during the first 2 h of machine perfusion inversely correlated with the perfusate oxygen concentration in a pig kidney autotransplant model and correlated with initial graft function [49]. Also FMN determination in the perfusate of NMP-kidneys has the potential to predict posttransplant renal function as recently published by the Cambridge group [94]. The value of FMN as perfusate biomarker during HMP (but also NMP) to evaluate transplantability and early graft function needs to be further explored in the context of human kidney transplantation.

## 8. When to Start Active Oxygenation during HMP?

In contrast to NMP where metabolic demands mandate oxygen is needed during the entire period of perfusion, it is less clear whether oxygenation is required during the total duration of HMP. Indeed, reducing the duration of oxygen delivery to a short period, either following organ retrieval or prior to implantation could confer significant logistical benefits (no need of oxygen source during organ transport and easier transport by airplane), independent of the oxygenation technique. We have previously demonstrated in a porcine DCD autotransplant model that brief oxygenation (2 h of the in total 22 h of preservation) at the start of HMP results in similar mitochondrial protection and initial graft function as compared with continuous (22 h of the in total 22 h of preservation) administration of oxygen during HMP [63]. The rationale for oxygen administration only at the start of HMP is to correct oxygen debt related to preceding warm ischemia before organ procurement (more pronounced in DCD than in DBD donors). The oxygen and ATP debt is usually corrected after 1 or 2 h after the start of active oxygenated HMP. In addition, machine-perfused kidneys are faster cooled below 8 °C because of peripheral vasodilatation and therefore reducing faster their residual metabolism as compared to SCS-preserved kidneys. DCD kidneys probably benefit more from this brief O_2_ uploading strategy as compared with DBD kidneys. However, the impact of warm ischemia in DBD kidneys is quite often underestimate and results often from the long time between start of in vivo cold perfusion and the final removal of the kidneys, especially when thoracic organs are procured before. Therefore, even DBD kidneys could benefit with this strategy.

## 9. Prospects: When to Start (Oxygenated) Machine Perfusion?

Independent of the organ preservation temperature, the optimal start time of machine perfusion (initial (on the procurement site)–continuous–at the end of preservation (at the recipient center)) depends mainly on the aim of its clinical application, in particular, preservation, organ quality assessment or organ repair. Figure 3 summarizes the wide range of machine perfusion strategies as described in recent publications [6,13,87,95]. NMP as an assessment tool has probably a wider range of options as compared with HMP(±O_2_), at the donor hospital, recipient center or for the whole time of organ preservation [13]. Multiple reasons explain the current limited implementation of NMP in clinical practice: technically complex, time-consuming, risk of organ loss in case of technical failure, the high cost of NMP (disposables, perfusate components, analyses for viability assessment), the need of a permanent availability of a dedicated staff (surgeons/perfusionists) and a specialized perfusion room. Results of the multicenter UK trial [18]. will be available soon and pilot projects are ongoing in the Netherlands and Canada.

Based on the above-mentioned clinical trials, oxygenated HMP used as an end-ischemic preservation strategy after SCS does not improve early kidney graft function and survival in DBD donors [47,51,77]. This result is in line with the Essen trial by Gallinat et al. demonstrating no benefit on early graft function using HMP without active oxygenation as an end ischemic preservation strategy after a period of SCS [96]. Both results might be explained by the fact that a brief period of HMP with or without active oxygenation after preceding SCS does not succeed to significantly decrease the ischemic-accumulated mitochondrial succinate responsible for a major part of the subsequent IRI. Continuous HMPO_2_ applied from the moment of procurement until transplantation seems to be feasible and safe and demonstrated positive effects on early kidney graft function and survival compared with continuous HMP without active oxygenation in DCD donors [50]. 

**Figure 4:**
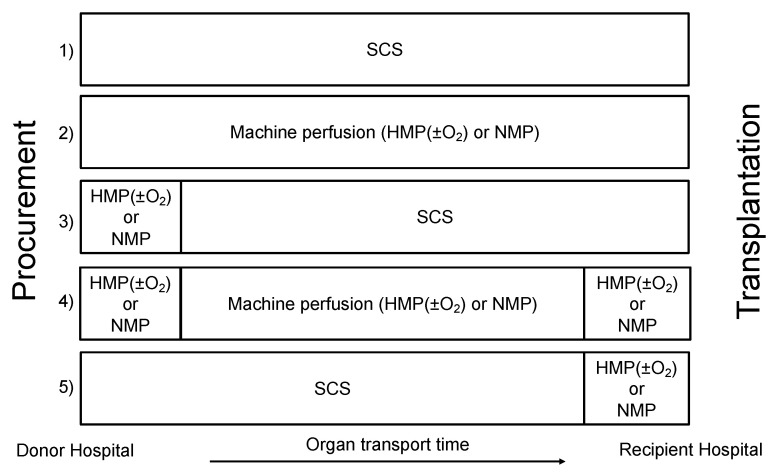
Overview of different machine perfusion strategies as compared to static cold storage alone (strategy 1). Strategy 2 consist of the same machine preservation strategy from immediately after procurement until transplantation. Strategy 3 and 4 include machine perfusion as assessment strategy at the donor hospital before accepting the organ for transplantation. Strategy 4 and 5 include machine perfusion assessment strategy and/or reconditioning strategy at the end of the preservation time after machine perfusion or SCS.

One of the future challenges in kidney will be to define which preservation strategy (HMP, NMP, SCS, subnormothermic machine perfusion, gradually controlled rewarming, …) or combination of different perfusion strategies (see Figure 4) is the most beneficial and it is likely that this will vary according to the donor type. Important factors to define these strategies will be the aim of its clinical application (improving organ preservation versus quality assessment or repair), and the difficulties to introduce machine perfusion on a national/international level, mostly because of logistical factors and resource restrictions.

## 10. Conclusions

Active oxygenation during HMP has been demonstrated to be feasible, safe and easy to implement in clinical practice. Based on current scientific evidence, oxygenated HMP is seemingly most beneficial in DCD kidneys when applied from the point of kidney procurement until transplantation as a strategy to improve preservation and subsequent early graft function. End ischemic application of this strategy after preceding SCS does not appear to offer any benefit for early graft function after ECD DBD or DCD donation [51]. The mitochondrial and immunological effect of oxygen during cold machine perfusion on early and late kidney graft function needs to be further explored and confirmed. Bubble and intermittent surface oxygenation is a promising technique and easy to implement in clinical practice to oxygenate organs by eliminating the cost of a membrane oxygenator and external oxygen source but feasibility needs to be confirmed in clinical trials.

## Figures and Tables

**Table 1:** Overview of pig studies investigating the effect of oxygenated HMP strategies of kidneys using an ex vivo normothermic reperfusion or an autotransplant model.

	Author, Year of Publication	Model	Study Groups (Number per Group)	PaO_2_, mmHg at 37 °C	HMP Strategy + Technique of O_2_	Main Conclusions
Ex Vivo Normothermic Reperfusion model	Koetting M et al., 2009 [75]	No WI + minimum 18 h preservation + 90 min ex vivo reperfusion	1. 18 h SCS (*n* = 6) 2. 18 h SCS + 2 h HMP (*n* = 6) 3. 18 h SCS + 2 h HMP 100% 02 (*n* = 6)	- NA ≥300	End ischemic Membrane oxygenation	Threefold improvement in renal creatinine clearance using 2 h end ischemic HMPO_2._
	Hoyer DP et al., 2014 [34]	30 min WI + 21 h preservation + 120 min ex vivo reperfusion	1. 21 h HMP 0% O_2_ (*n* = 5) 2. 21 h HMP air (*n* = 5) 3. 21 h HMP 100% O_2_ (*n* = 5)	0 200–220 760–800	Continuous Membrane oxygenation	HMP 100% O_2_ provides the best initial graft function, followed by HMP 20% O_2_ and HMP 0% O_2_.
	Venema LH et al., 2018 [72]	30 min WI + 24 h preservation + 240 min ex vivo reperfusion	1. 24 h SCS (n = 6) 2. 24 h HMP 0% O_2_ (*n* = 6) 3. 24 h HMP 21% O_2_ (*n* = 6) 4. 24 h HMP 100% O_2_ (n = 6)	- 0 NA NA	Continuous Membrane oxygenation	All HMP groups significantly better than SCS group. No difference between all HMP groups.
Autotransplant model	Gallinat A et al., 2012 [73]	No WI + 21 h preservation	1. 21 h HMP 0% O_2_ (*n* = 5) 2. 21 h HMP 100% O_2_ (*n* = 5)	0 >500	Continuous Membrane oxygenation	Kidney function significantly better after HMP 0% O_2_ compared to HMP 100% O_2_.
	Thuillier R et al., 2013 [74]	60 min WI + 22 h preservation	1. 22 h HMP 0% O_2_ (*n* = 4) 2. 22 h HMP 100% O_2_ (*n* = 4)	0NA	Continuous Membrane oxygenation	HMP100% O_2_ lower serum creatinine peak and faster return to normal levels compared to HMP 0% O_2_.
	Kasil A et al., 2019 [76]	60 min WI + 23 h preservation	1. 23 h HMP (*n* = 6) 2. 23 h HMP 100% O_2_ (*n* = 6) 3. 23 h HMP + M101 2 g/L (*n* = 6) 4. 23 h HMP 100% O_2_ + M101 2 g/L (*n* = 6)	NA NA NA NA	Continuous Membrane oxygenation	Supplementation with hemoglobin of the marine worm (M101) associated with or without 100% O_2_ improved the machine perfusion effect upon kidney recovery and late graft outcome.
	Darius T et al., 2020 [49]	30 min WI + 22 h preservation	1. 22 h SCS (*n* = 6) 2. 22 h HMP (*n* = 6) 3. 22 h HMPO_2_low (*n* = 8) 4. 22 h HMPO_2_high (*n* = 8)	- 65–75 210–230 680–760	Continuous Membrane oxygenation	HMPO_2_low and HMPO_2_high demonstrated significant better initial graft function compared to HMP without active oxygenation. All HMP groups demonstrated significant better initial graft function compared to SCS.
	Darius T et al., 2020 [63]	30 min WI + 22 h preservation	1. 22 h HMP (n = 6) 2. 22 h HMPO_2_ (n = 8) 3. 2 h HMPO_2_ + 20 h HMP (n = 6) 4. 20 h HMP + 2 h HMPO_2_	- 680–760 700→250 250→700	Continuous Membrane oxygenation	The brief initial and continuous oxygenated HMP groups were associated with superior graft recovery compared to either HMP without active oxygenation or kidneys oxygenated at the end of HMP.
	Darius T et al., 2020 [58]	30 min WI + 22 h preservation	1. 22 h HMP + intermittent surface oxygenation (30 min at start HMP and 60 min at end HMP) (*). 2. 22 h standard HMP (n = 6) 3. 22 h HMPO_2_ (n = 8) (**) 4. 2 h HMPO_2_ + 20 h HMP (n = 6) (**) Remarque: group 2–4 are historical control groups.	>00 during the first 2 h of HMP 65–75 680–760 700→250	Continuous Bubble and surface oxygenation (*) versus membrane oxygenation (**)	Brief bubble and 30 min surface oxygenation of the perfusate effectively maintained supraphysiological PaO_2_ levels during the first 2 h of HMP with improved flow dynamics. Both oxygenation techniques yielded similar, superior early graft function compared with HMP without active oxygenation.

**Table 2:** Overview of clinical studies using oxygenated hypothermic machine perfusion for kidney preservation.

	Meister FA et al., 2019 [77]	Ravaioli M et al., 2020 [47]	Jochmans I et al., 2020 [50]	Husen P et al., 2021 [51]	Houtzager J et al., 2021 [48]
Donor type	ECD DBD	ECD DBD	DCD > 50y	ECD DBD	DBD DCD
Perfusion device	Kidney Assist (Organ Assist)	Unique device developed by Medica S.P.A and Centro Iperbarico S.R.L.	Kidney Assist (Organ Assist)	Kidney Assist (Organ Assist)	Oxygenated Airdrive HMP system
Type of study	Matched-case analysis	Matched-case analysis	RCT (COMPARE trial)	RCT (POMP trial)	Phase I
Study groups (*n* = included patients)	1. SCS + HMPO_2_ (*n* = 15) 2. SCS (*n* = 30) (historical cohort group)	1. SCS + HMPO_2_ (*n* = 10) 2. SCS (*n* = 30) (historical cohort group)	1. HMPO_2_ (*n* = 106) 2. HMP (*n* = 106)	1. SCS + HMPO_2_ (*n* = 127) 2. SCS (*n* = 135)	1.SCS (*n* = 4)/HMP (*n* = 1) + HMPO_2_
Donor age, year					
Study group 1	66 (±12) *	71.5 (60–78)	58.0 (54.0–63.0)	64.0 (50.0–82.0)	44.2 (19–64)
Study group 2	66 (±8) *	69.5 (59–79)	58.0 (54.0–63.0)	65.0 (51.0–84.0)	
Donor warm ischaemia time, min					
Study group 1	-	-	28.8 (22–36)	34.0 (17.0–92.0)	NA
Study group 2	-	-	28.8 (22–36)	32.0 (11.0–80.0)	NA
Duration on MP, hour					
Study group 1	152 ± 92 min *	Mean 3.3 (1–6 h)	6.85 (4.5–9.1)	4.67 (0.8–17.1)	8.5 (3–15)
Study group 2	/	/	7.40 (4.8–9.9)	/	
*p* value			0.21		
Cold ischemia time, hour					
Study group 1	646 ± 227 *	14.5 (10.8–22)	11.0 (8.7–13.7)	13.2 (5.1–28.7)	20.2 (11–29.5)
Study group 2	674 ± 214 *	14 (8–21)	10.3 (8.9–14.0)	12.9 (4–29.2)	
*p* value	0.563	0.896	0.41		
DGF rate, n (%)					
Study group 1	8 (53%)	2 (20%)	38 (36%)	30 (23.6%)	3 (60%)
Study group 2	10 (33%)	12 (40%)	38 (36%)	38 (28.1%)	
*p* value	0.197	0.607	0.99	0.40	
Functional DGF rate	NA	NA			
Study group 1			76 (72%)	76 (59.8%)	NA
Study group 2			76 (72%)	93 (68.9%)	NA
*p* value			0.99	0.13	
PNF rate, n (%)					
Study group 1	1 (7%)	0 (0%)	3 (3%)	8 (6.3%)	1 (20%)
Study group 2	0 (0%)	1 (3.3%)	5 (5%)	8 (5.9%)	
*p* value	0.333	0.948	0.48	0.90	
eGFR	(at 6 mo)	NA	(at 1 year)	(at 1 year)	NA
Study group 1	32 ± 14 *		50.5 ± 19.3	39.9 (14.4)	
Study group 2	38 ± 17 *		46.7 ± 17.1	41.2 (17.1)	
*p* value	0.276		0.12	0.53	
Graft survival, %	(at 6 months)	(at 1 year)	(at 1 year)	(at 1 year)	NA
Study group 1	93%	100%	10%	92.1%	
Study group 2	100%	93.3%	90%	93.3%	
*p* value	0.333	0.894	0.028	0.63	
Postoperative complications, % (Clavien-Dindo grade 3 or more)					
Study group 1			11%		
Study group 2	NA	NA	13%	NA	NA
*p* value			*p* = 0.032		
BPAR	NA	NA			NA
Study group 1			15 (14%)	23 (18.1%)	
Study group 2			27 (26%)	18 (13.3%)	
*p* value			0.040	0.29	

Data are presented as median (range), except *.

## Data Availability

Not applicable.

## Abbreviations

ADP, adenosine diphosphate; AOR, adjusted odds ratio; ATP, adenosine triphosphate; BPAR, biopsy proven acute rejection; CI-CIV, mitochondrial complex 1–4; CAC, citric acid cycle; DBD, donation after brain death; DCD, donation after circulatory death; DGF, delayed graft function; ECD, expanded criteria donors; eGFR, estimated glomerular filtration rate; FET, forward electron transport; FMN, flavin mononucleotide; FMNH_2_, reduced flavin; HMP, hypothermic machine perfusion; HMPO2, oxygenated hypothermic machine perfusion; NA, not available; NADH, reduced nicotinamide adeninedinucleotide; NMP, normothermic machine perfusion; M101, hemoglobin of the marine worm; O_2_, oxygen; PaO2, partial oxygen pressure measured via arterial line; pO_2_, partial oxygen pressure; PNF, primary nonfunction; IRI, ischemia-reperfusion injury; Q, ubiquinone; QH2, ubiquinol; RCT, randomized clinical trial; RET, reverse electron transport; ROS, reactive oxygen species; SCS, static cold storage; WI, warm ischemia.
